# Influenza‐like illness‐related emergency department visits: Christmas and New Year holiday peaks and relationships with laboratory‐confirmed respiratory virus detections, Edmonton, Alberta, 2004–2014

**DOI:** 10.1111/irv.12416

**Published:** 2016-08-30

**Authors:** Leah J. Martin, Cindy Im, Huiru Dong, Bonita E. Lee, James Talbot, David P. Meurer, Shamir N. Mukhi, Steven J. Drews, Yutaka Yasui

**Affiliations:** ^1^ School of Public HealthUniversity of AlbertaEdmontonABCanada; ^2^ Department of PediatricsUniversity of AlbertaEdmontonABCanada; ^3^ Office of the Chief Medical Officer of HealthAlberta HealthEdmontonABCanada; ^4^ Alberta Health ServicesEdmontonABCanada; ^5^ Canadian Network for Public Health IntelligenceNational Microbiology LaboratoryWinnipegMBCanada; ^6^ Provincial Laboratory for Public Health (ProvLab) AlbertaAlberta Health ServicesEdmontonABCanada; ^7^ Department of Laboratory Medicine and PathologyUniversity of AlbertaEdmontonABCanada; ^8^ Department of Epidemiology and Cancer ControlSt. Jude Children's Research HospitalMemphisTNUSA

**Keywords:** Emergency Medical Services, influenza, respiratory syncytial viruses, respiratory tract infections, surveillance

## Abstract

**Background:**

Emergency department (ED) visit volumes can be especially high during the Christmas–New Year holidays, a period occurring during the influenza season in Canada.

**Methods:**

Using daily data, we examined the relationship between ED visits for the chief complaint “cough” (for Edmonton, Alberta residents) and laboratory detections for influenza A and respiratory syncytial virus (RSV) (for Edmonton and surrounding areas), lagged 0–5 days ahead, for non‐pandemic years (2004–2008 and 2010–2014) using multivariable linear regression adjusting for temporal variables. We defined these cough‐related visits as influenza‐like illness (ILI)‐related ED visits and, for 2004–2014, compared Christmas–New Year holiday (December 24–January 3) and non‐holiday volumes during the influenza season (October–April).

**Results:**

Adjusting for temporal variables, ILI‐related ED visits were significantly associated with laboratory detections for influenza A and RSV. During non‐pandemic years, the highest peak in ILI‐related visit volumes always occurred during the holidays. The median number of holiday ILI‐related visits/day (42.5) was almost twice the non‐holiday median (24) and was even higher in 2012–2013 (80) and 2013–2014 (86). Holiday ILI‐related ED visit volumes/100 000 population ranged from 56.0 (2010–2011) to 117.4 (2012–2013). In contrast, lower visit volumes occurred during the holidays of pandemic‐affected years (2008–2010).

**Conclusions:**

During non‐pandemic years, ILI‐related ED visit volumes were associated with variations in detections for influenza A and RSV and always peaked during the Christmas–New Year holidays. This predictability should be used to prepare for, and possibly prevent, this increase in healthcare use; however, interventions beyond disease prevention strategies are likely needed.

## 1 Introduction

Emergency departments (EDs) can experience especially high visit volumes during the Christmas and New Year holidays.^1^ In Canada, this surge has been highlighted by the media^2^, ^3^ and by hospitals themselves^4^ and, because this holiday period occurs during the influenza season, it is important to consider the contribution that influenza‐like illness (ILI) makes to the increase occurring at this time. For example, at one hospital in Winnipeg, Manitoba, Canada, between 1995 and 1999, ED visits related to ILI increased by as much as 61% (128 visits) during the Christmas holidays compared with the week before Christmas.^5^ Aside from EDs, in Canada, individuals experiencing ILI‐related symptoms may also visit family physicians, whose offices may be closed during the Christmas holidays, or walk‐in clinics.

The influenza season of 2012–2013 was especially intense in Canada^6^ and, in January of the following season (2013–2014), an influenza care clinic was opened in Edmonton, Alberta to reduce ED visit volumes following the Christmas–New Year holidays.^7^ Quantifying ILI‐related ED visit volumes during these comparatively severe influenza seasons and during the Christmas–New Year holidays in particular may improve planning and preparedness for future surges in ILI‐related ED use.

We investigated ILI‐related ED visits in Edmonton, Alberta, quantifying the magnitude and duration of peak volumes, noting the dates when peak volumes were highest, and compared volumes during the Christmas–New Year holiday period to those during the remainder of the influenza season. To evaluate our definition of ILI, we first examined the relationship between these ILI‐related ED visits and laboratory detections for respiratory viruses in Edmonton and surrounding geographic areas.

## 2 Methods

### 2.1 Overview

Our study is divided into two parts. In Part 1, we evaluate our definition of ILI‐related ED visits by examining the relationship between ED visits for the chief complaint “cough” and laboratory‐confirmed respiratory virus detections. In Part 2, we explore these ILI‐related ED visit volumes in more depth, investigating the timing and magnitude of surges in volume during the Christmas–New Year holiday and non‐holiday periods, similar to others who have looked at total ED visits.^1^ Overall, we include a total of 3423 days of data (3 October 2004 to 15 February 2014). We defined each annual season as week 40 of one calendar year to week 39 of the next calendar year, based on the weeks from the Centers for Disease Control and Prevention^8^ (note: 2013–2014 data ended at week 7); therefore, we include nine complete and one partial annual season. We defined the 2008–2009 and 2009–2010 seasons (28 September 2008 to 2 October 2010) as “pandemic‐affected” and the remaining seasons (2004–2008 and 2010–2014) as “non‐pandemic” seasons. We limit Part 1 to non‐pandemic seasons (n=2688 days) while, in Part 2, we include the entire study period but focus our analysis on the influenza season. We defined a consistent Christmas–New Year holiday period from season‐to‐season: December 24 of one calendar year to January 3 of the following calendar year (11 days); this is similar to a study by Zheng et al.,^1^ but they used a longer, 28‐day period.

### 2.2 Emergency department visit data

To quantify ILI‐related healthcare use, we used daily data from the Alberta Real Time Syndromic Surveillance Net (ARTSSN), which provides syndromic surveillance data for Edmonton, Alberta, and surrounding areas^9^ and included data from nine EDs, three of which began contributing data after the study began (in 2004, 2005 and 2013). ARTSSN data included the visit date and chief complaint, as well as the patients' demographic information (age and sex) and area of residence. We included ED visits that listed chief complaints for “cough”. Originally, we investigated ILI‐related visits with chief complaints of “fever” and “sore throat”; however, these were excluded from further analysis because preliminary analyses demonstrated weaker relationships between these chief complaints and respiratory virus detections in our preliminary analyses (Pearson correlation coefficients for influenza A and RSV, respectively, were 0.35 and 0.27 for fever and 0.10 and 0.058 for sore throat). This may be due, in part, to the way in which chief complaint data are entered into the system: only one chief complaint is allowed and it is meant to be the most prominent symptom. We limited our analysis to ED visits made by residents of the city of Edmonton, based on the forward sortation area (FSA) of their postal code (n=38 FSAs with a total population of 815 812 in 2011^10^) in order to calculate rates in Part 2. We included all ILI‐related ED visits regardless of discharge disposition (i.e. we included visits categorized as “left without being seen”). For context, Edmonton residents made a total of approximately 2.5 million ED visits for any chief complaint during the study period according to the ARTSSN data.

### 2.3 Respiratory virus detection data

From the Provincial Laboratory for Public Health (ProvLab), Alberta, we accessed daily respiratory virus detections data for influenza A, influenza B, parainfluenza, adenoviruses and respiratory syncytial virus (RSV) using Data Integration for Alberta Laboratories (DIAL).^11^ ProvLab conducts laboratory testing for respiratory viruses for the province of Alberta and neighbouring territories. During the study, detection of respiratory viruses was performed using various algorithms: 2004–2005 (direct fluorescent antibodies test (DFA) and/or viral cultures), 2005–2008 (DFA and/or “in‐house” nucleic acid amplification tests [NAT]), 2008–2014 (DFA and/or NAT and/or xTAG respiratory viral panel classic assay [Luminex Molecular Diagnostics, Toronto, ON, Canada]) as described previously.^12^, ^13^, ^14^ For each positive laboratory sample, we used the date of receipt in the analyses and defined the geographic region using a hierarchy of information as provided for the patient (city of residence), physician (location of practice in laboratory information system) and submitting location (e.g. medical clinic or hospital) for the specimen (in descending order). For example, if the patient's region was unavailable, we used the physician's region and if this was also unavailable, we used the region of the submitting location. Based on this definition, we limited our analysis to samples from Region 6, which is the broad geographic area that includes Edmonton (see map: http://www.ahvna.org/pdfs/RHA-Map-December-1-2003.pdf).

### 2.4 Part 1: comparing laboratory detections with ILI‐related ED visits

For Part 1, we used ARTSSN data (aggregated by patient age and sex) and laboratory data to compare the relationships between the following time series: daily ILI‐related ED visit counts and daily laboratory detections for each of the respiratory viruses. We omitted pandemic‐affected seasons from Part 1 so that changes in either the laboratory testing procedures or the healthcare seeking behaviour of the population would not distort the overall relationship observed between these two datasets.

We examined the number of laboratory detections for each virus and the variation in detections over time. To compare laboratory data and ED visit data, we began with a descriptive analysis examining joint time‐series plots of the number of detections for each of the respiratory viruses and ILI‐related ED visits and calculated associated Pearson's correlation coefficients. We used these descriptive statistics as a rationale to decide which respiratory viruses to include in the remainder of the analysis. Then, we investigated lags for each of the respiratory viruses; that is, we allowed the laboratory detections to either precede or follow ED visits. We investigated lags from −20 days to +20 days, similar to van den Wijngaard et al.^15^ who examined lags of ±5 weeks. However, our final models used lags of 0–5 days (where laboratory counts followed ED visits) for several reasons, including the: (i) short expected lag time between a visit to the ED and receipt of a laboratory sample, (ii) understanding that the receipt of a laboratory sample would likely follow a healthcare visit and (iii) observed correlations. These lags removed a total of 10 days from the lagged analyses in Part 1: five days at the end of the data set and an additional five days across the break in the data due to the H1N1 pandemic because we did not lag the laboratory data between the 2007–2008 and 2010–2011 seasons. To compare the peak timing of laboratory detections to ILI‐related ED visits, we also considered an “extended” Christmas–New Year holiday period as December 24 to January 8 of the following calendar year (16 days), which allowed the five‐day lag to be considered.

To characterize variations in daily ILI‐related ED visits with respect to respiratory virus detections, similar to van den Wijngaard et al.,^15^ we examined multivariable ordinary least squares regression models and adjusted for seasonality using two sinusoidal terms, $\text{sin}(\frac{2\pi }{365.25}\text{day})$ and $\text{cos}(\frac{2\pi }{365.25}\text{day})$ (where day=1, 2, 3, …, 2688) for the n=2688 days included in Part 1. After examining residuals, we adjusted for long‐term temporal characteristics of the ILI‐related ED visit data using a linear time trend. Additionally, we adjusted for indicator variables for each day of the week and the 11‐day Christmas–New Year holiday period. In our final model, we included all lags (0–5 days) for each respiratory virus that we considered for further investigations and all temporal adjustment variables. We examined regression parameter estimates and assessed *R* ^2^. To examine autocorrelation of residuals after model fitting, we used the Durbin–Watson test and added two autoregressive terms to address first‐order autocorrelation in our final model. For comparison, we reran our final model for each lagged respiratory virus separately. Bhaskaran et al.^16^ provide an overview of time‐series analysis that was helpful in our study.

### 2.5 Part 2: examining ILI‐related ED visits

We compared ILI‐related ED visit volumes during the Christmas–New Year holiday period to those during the rest of the influenza season (October–April). To allow comparison of the visit volume over the study period given the increases in population of the city, we calculated ILI‐related ED visits per 100 000 population. Using data from the 2001–2011 Canadian censuses,^10^, ^17^, ^18^ we interpolated and extrapolated the population of the FSAs included in the study. We defined a high volume day as one with at least 5.4 ILI‐related visits per 100 000 population (the 95th percentile of the ILI‐related ED visit rate during non‐pandemic seasons) and examined the frequency of high volume days and the number of consecutive high volume days throughout the year. For Part 2, we included data from the 2009 H1N1 pandemic, but we examined the pandemic‐affected and non‐pandemic seasons separately.

We received ethics approval from the University of Alberta Health Research Ethics Board. Analyses were conducted using sas 9.4 (SAS Institute Inc., Cary, North Carolina) and r version 3.1.2.^19^

## 3 Results

### 3.1 Part 1

#### 3.1.1 Descriptive analyses

During non‐pandemic seasons, 14 955 laboratory samples tested positive for at least one of the respiratory viruses considered, representing 15 262 detections, with the highest number of detections for RSV (40%) and influenza A (26%) (Table 1). Of the 306 mixed virus samples, 11 viral combinations were seen, most commonly parainfluenza–adenovirus (n=87), RSV–adenovirus (n=73), and RSV–parainfluenza (n=63). A submitting location was provided for 91% of samples, of which most were acute care hospitals and non‐urban health centres (n=11 381, 83%). These samples would have been submitted from inpatient wards, EDs, as well as hospital‐based clinics. Compared with other viruses, influenza detections (especially influenza A) were associated with an older median patient age (Table 1). Overall, approximately half the laboratory detections were from female patients, but this proportion was somewhat lower for adenovirus (39%) (Table 1). During these non‐pandemic seasons, Edmonton residents made 59 832 ILI‐related ED visits (Table 1).

**Table 1:** Characteristics of influenza‐like illness (ILI)‐related emergency department (ED) visits and respiratory virus laboratory detections, including Pearson correlation coefficients, during non‐pandemic seasons, 2004–2008 and 2010–2014, Edmonton, Alberta and surrounding area

Chief complaint or respiratory virus	No. (%)	Median no. per day^a^(SD, range)	Patient age (years, median, IQR)	Patient sex (No. female, %)	Correlation with ILI‐related ED visits^f^
ILI‐related ED visits					
Cough	59 832	20 (12.2, 1–125)	8 (1–41)	28 719 (48%)	–
Laboratory detections					
RSV	5943 (40%)	3 (4.1, 1–29)	0 (0–2)	2714 (46%)	.47
Influenza A	3813 (26%)	2 (5.4, 1–57)	37 (6–63)^c^	1924 (51%)^c^	.61
Parainfluenza	2910 (19%)	1 (1.3, 1–21)	2 (0–18)^c^	1301 (45%)	.18
Adenovirus	1372 (9%)	1 (0.8, 1–5)	1 (0–4)^c^	529 (39%)^c^	.04
Influenza B	1224 (8%)	2 (1.8, 1–12)	13 (4–48)^c^	598 (49%)^d^	.21
Overall	15 262^b^	5 (7.2, 1–64)	2 (0–35)^e^	7066 (47%)^e^	–

a Excludes days with zero detections for the respiratory virus(es) in question.

b From n=14 955 samples: 305 mixed samples contributed two detections each and one mixed sample contributed three detections.

c *P*<.001 compared to detections for RSV (Wilcoxon Rank Sum test for age and chi‐square test for sex).

d *P*<.05 compared to detections for RSV (chi‐square test).

e n=38 detections were missing patient age and n=123 detections were missing patient sex.

f All are statistically significant at *P*<.0001 except adenovirus at *P*<.05.

ILI, influenza‐like illness; RSV, respiratory syncytial virus.

Comparing the viruses to each other, influenza A and RSV had the highest number of detections, highest variation in the number of detections, and the strongest, unlagged, unadjusted correlations with ILI‐related ED visits (Table 1). For these reasons, and because our goal was to investigate whether ILI‐related ED visits were associated with respiratory virus detections rather than how each virus might help to explain variation in visits, we decided to focus the rest of our analysis on influenza A and RSV.

#### 3.1.2 Relationships between ILI‐related ED visits and viral detections

**Figure 1:**
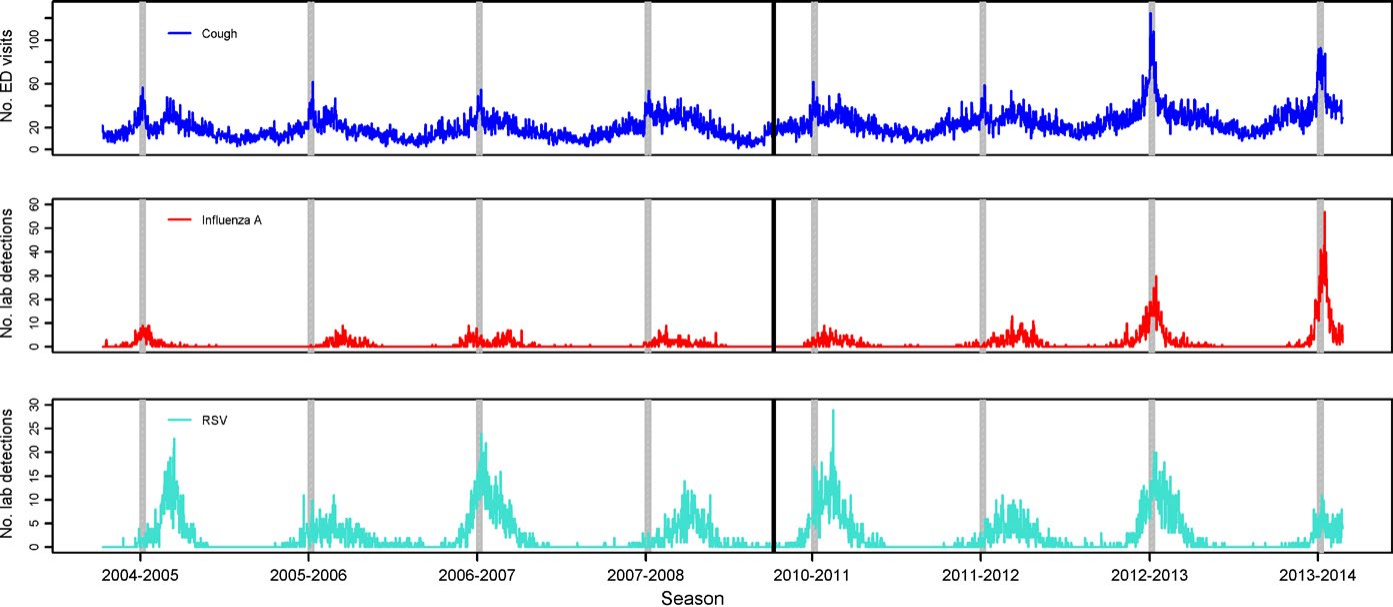
Comparison of daily influenza‐like illness (ILI)‐related emergency department (ED) visits for cough and laboratory detections for influenza A and respiratory syncytial virus (RSV) during non‐pandemic seasons. Black vertical lines divide the time periods before and after the 2009 H1N1 pandemic period. Grey vertical bars mark the 11‐day Christmas‐New Year holiday period for each season.

We visually compared ILI‐related visit volumes and respiratory virus detections over time (Fig. 1). In 2004–2005, two distinct peaks in ILI‐related ED visits can be seen, which correspond well with separate peaks for influenza A followed by RSV (Fig. 1). Although the maximum peak in ILI‐related ED visit volumes always occurred during the Christmas–New Year holidays (see Part 2 for more details), the timing of maximum peaks in respiratory virus detections varied from season‐to‐season. However, considering RSV and influenza A together, during four of the eight non‐pandemic seasons (2004–2005, 2006–2007, 2012–2013 and 2013–2014), either influenza A, RSV detections or both had a maximum peak during the extended holiday period (24 December–8 January). In 2012–2013, the maximum peak in detections for both of these viruses occurred during the extended holiday period and the number of detections was relatively high; this corresponds to the non‐pandemic season with the highest volume of ILI‐related ED visits (Fig. 1). In the next season, 2013–2014, influenza A peaked during the extended holiday period to the highest level throughout the study period in Part 1 (i.e. non‐pandemic seasons) and RSV also had a small peak during this time; this corresponds to the non‐pandemic season with the second‐highest volume of ILI‐related ED visits (Fig. 1).

#### 3.1.3 Multivariable analysis

Our final temporally adjusted multivariable linear regression model with two autoregressive terms explained an estimated 77% of the variability in ILI‐related ED visits (Table 2). Visits were highest on Sundays and during the Christmas–New Year holidays, which was associated with an adjusted estimated increase of 14.0 (95% CI=11.8–16.1) visits compared with the rest of the year. Additive parameter estimates over the six lags (for 0–5 days) were 1.2 for influenza A and 0.4 for RSV (Table 2). Therefore, an increase in one influenza A detection on each of these six days was associated with an increase of 1.2 ILI‐related ED visits on day 0. In comparison, an increase in one RSV detection on each of these six days was associated with an increase of only 0.4 ILI‐related ED visits on day 0. The statistically significant lags were days 1–4 for influenza A and days 1–2 for RSV (Table 2); these lags represent an estimate of the time delay between ILI‐related ED visits and receipt of positive tests at the laboratory. Of note, ED visits for some days within the Christmas–New Year holidays in 2012–2013 and 2013–2014 were not well fit by this model. In particular, the n=125 visits on 26 December 2012 had a large positive residual. For comparison, we reran the final model separately for lagged influenza A and for lagged RSV; compared with the final model, the additive estimate was similar for influenza A (1.3, *R* ^2^=.76) and somewhat higher for RSV (0.7, *R* ^2^=.74).

**Table 2:** Model for influenza‐like illness (ILI)‐related emergency department (ED) visits for chief complaint cough and respiratory viruses, non‐pandemic seasons, 2004–2014, Edmonton, Alberta and surrounding areas (Total *R* ^2 ^= .77)^a^

Variable		Parameter estimate	95% CI		*P*‐value
Intercept		−34.95	−41.95	−27.95	<0.0001
Temporal adjustment variables					
Sine		5.26	4.51	6.01	<0.0001
Cosine		−2.04	−2.70	−1.38	<0.0001
Date		0.003	0.003	0.004	<0.0001
Christmas‐New Year holiday (Dec 24‐Jan 3)		13.95	11.80	16.09	<0.0001
Sunday (ref)		1.00	−	−	−
Monday		−2.29	−3.06	−1.51	<0.0001
Tuesday		−4.29	−5.10	−3.48	<0.0001
Wednesday		−4.71	−5.57	−3.84	<0.0001
Thursday		−6.01	−6.88	−5.14	<0.0001
Friday		−5.54	−6.36	−4.73	<0.0001
Saturday		−3.31	−4.08	−2.53	<0.0001
Virus	Lag (days)				
Influenza A^b^	0	−0.02	−0.15	0.12	0.79
	1	0.35	0.22	0.49	<0.0001
	2	0.34	0.21	0.47	<0.0001
	3	0.33	0.20	0.46	<0.0001
	4	0.20	0.069	0.34	0.0021
	5	0.03	−0.10	0.17	0.62
RSV^c^	0	0.09	−0.029	0.21	0.12
	1	0.14	0.023	0.26	0.015
	2	0.19	0.078	0.31	0.0007
	3	−0.04	−0.16	0.08	0.49
	4	−0.02	−0.14	0.10	0.77
	5	0.02	−0.10	0.14	0.77

a Two autoregressive terms were added to this model.

b Additive estimate = 1.24; Significant lags = day 1–4.

c Additive estimate =0.39; Significant lags = day 1–2.

### 3.2 Part 2

During non‐pandemic and pandemic seasons combined, Edmonton residents made 79 431 ED visits for the chief complaint “cough”: 57 660 during influenza seasons and 5257 specifically during the Christmas–New Year holiday periods. The majority of these visits, both during the holidays (58%) and the remainder of the influenza season (59%), were made to two large healthcare centres.

#### 3.2.1 Non‐pandemic seasons

**Figure 2:**
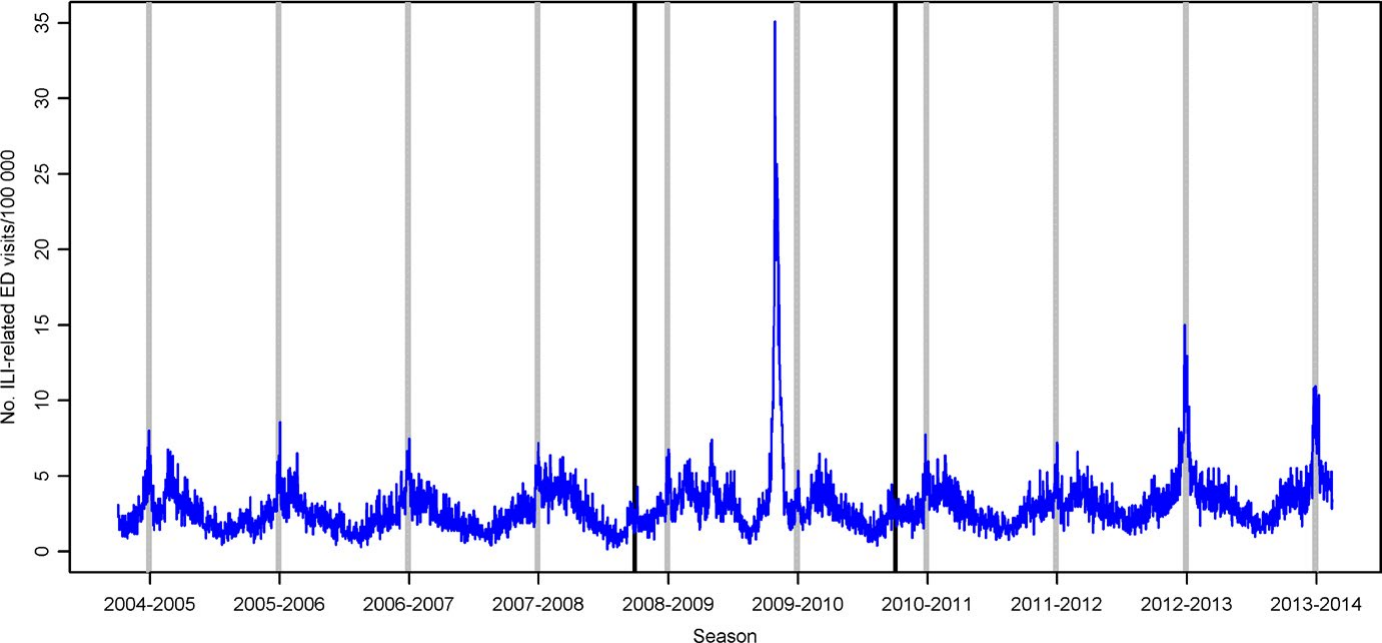
Daily number of influenza‐like illness (ILI)‐related emergency department (ED) visits per 100 000 population among Edmonton residents. Edmonton, Alberta, October 2004 ‐ February 2014. Grey vertical bars indicate the 11‐day Christmas‐New Year holiday period each season; black vertical lines indicate the beginning and end of the 2009 H1N1 pandemic period.

During each non‐pandemic season, the highest peak in daily ILI‐related ED visit volumes occurred during the Christmas–New Year holidays, specifically between December 26 and January 2 (Fig. 1, Table 3). Additionally, several seasons had comparable, but lower, peaks in late January to early March: 2004–2005, 2005–2006, 2007–2008, 2010–2011, and 2011–2012 (Fig. 2). The median number of daily ILI‐related visits during the holidays was almost double the median occurring during the remainder of the influenza season (42.5 vs 24 visits/day) (Table 3).

**Table 3:** Influenza‐like illness (ILI)‐related emergency department (ED) visits by Edmonton residents during holiday and non‐holiday periods, 2004–2014

Season	No. holiday ILI‐related visits(% of total ILI‐related visits, Oct–Apr)	No. holiday ILI‐related visits/100 000 population	Median no. daily ILI‐related visits (range)		Date of maximum ILI‐related visit volume (Oct–Apr)
			Christmas–New Year holidays (24 Dec‐3 Jan)	Non‐holiday portion of influenza season (Oct–Apr)	
Non‐pandemic seasons					
2004–2005	463 (10)	65.3	42 (27–57)	20 (6–48)	Dec 29/04
2005–2006	421 (9.8)	58.4	37 (28–62)	19 (4–47)	Jan 2/06
2006–2007	440 (9.5)	59.9	39 (32–55)	20 (6–39)	Jan 1/07
2007–2008	443 (7.9)	59.0	41 (30–54)	25 (7–48)	Dec 31/07
2010–2011	448 (8.2)	56.0	42 (27–62)	24 (7–51)	Dec 26/10
2011–2012	459 (7.8)	56.3	37 (29–59)	27 (9–54)	Jan 1/12
2012–2013	977 (13)	117.4	80 (65–125)	31 (13–80)	Dec 26/12
2013–2014	883 (–)^a^	104.1	86 (55–93)	35 (14–88)	Dec 29/13
Overall	4534 (9.5)^a^	73.0	42.5 (27–125)	24 (4–88)	–
Pandemic‐affected seasons					
2008–2009	391 (7.5)	51.0	35 (19–52)	22 (6–55)	Apr 30/09^b^Jan 1/09^c^
2009–2010	332 (3.8)	42.4	31 (21–42)	26 (11–275)	Oct 28/09^b^Mar 2/10^c^
Overall	723 (7.6)	46.7	32 (19–52)	24 (6–275)	–

a For 2013–2014, the season includes weeks 40 to 7 only; therefore, the holiday period represents a longer time period as a proportion of the overall season and the percentage of visits is not calculated or included in the overall percentage.

b During H1N1 pandemic: maxima of n=55 visits in 2008–2009 and n=275 visits in 2009–2010.

c During the non‐pandemic portion of the season: maxima of n=52 visits in 2008–2009 and n=51 in 2009–2010.

ILI, influenza‐like illness.

Of all non‐pandemic seasons in the study, 2012–2013 and 2013–2014 had the highest ILI‐related visit volumes, both over the influenza season as a whole and during the Christmas–New Year holidays specifically, with the highest peak volumes occurring on Boxing Day (a holiday in Canada) in 2012 (n=125 visits) and 29 December 2013 (n=93 visits) (Fig. 1, Table 3). For reference, this holiday maximum (n=125 visits in 2012) represents 45% of the highest peak ILI‐related ED visit volume during the second wave of H1N1 (n=275 visits on 28 October 2009).

Between 421 and 977 ILI‐related visits were made during the holidays in each season (Table 1), representing 56 to 65 ILI‐related ED visits/100 000 population during this 11‐day period in 2004–2012; however, in 2012–2013 and 2013–2014, this increased markedly to 104 and 117 ILI‐related ED visits/100 000 population, respectively (Table 1, Fig. 2).

Of the 1620 days during non‐pandemic influenza seasons, 135 were high volume days, of which 40% (n=54 days) occurred during the Christmas–New Year holidays, an 11‐day period representing only 5.4% of these 1620 days. Most high volume days occurred in 2012–2013 (n=44 days) and 2013–2014 (n=31 days) and the longest duration of consecutive high volume days also occurred during these seasons: 2012–2013 (n=24 days, 15 December to 7 January) and 2013–2014 (n=20 days, 21 December to 9 January), both overlapping the Christmas–New Year holidays.

#### 3.2.2 Pandemic‐affected seasons

In 2008–2009 and 2009–2010, ILI‐related visit volumes during the Christmas–New Year holidays were the lowest observed among any of the seasons examined, representing 47 visits/100 000 population (Table 3, Fig. 2). Similar to non‐pandemic seasons, the highest daily visit volume in 2008–2009, other than the first pandemic wave, occurred during the Christmas–New Year holidays on January 1, 2009 (n=52 visits; Table 3, Fig. 2). In contrast, the highest daily visit volume in 2009–2010, other than the second pandemic wave, occurred in March (Table 3, Fig. 2).

## 4 Discussion

We examined the relationships between ILI‐related ED visits and laboratory detections for respiratory viruses and examined ILI‐related ED visits during the Christmas–New Year holidays in more detail. After adjusting for a linear time trend, seasonality, the Christmas–New Year holidays and day of the week, ED visits for the chief complaint “cough” were associated with variations in laboratory detections for influenza A and RSV; we observed a stronger association for influenza A than for RSV. During non‐pandemic seasons, ILI‐related ED visits consistently peaked to their highest volume during the Christmas–New Year holidays, with the median number of daily visits almost doubling during the holidays compared with the rest of the influenza season. However, the timing of the highest peaks in laboratory detections was more variable. Compared with other seasons, 2012–2013 and 2013–2014 had the highest holiday ILI‐related ED visit volumes, in both absolute terms and proportional to the population, with 2012–2013 being the most intense. Both of these seasons also had the highest number of laboratory detections for influenza A out of any non‐pandemic season and, in 2012–2013, a simultaneous high number of RSV detections.

Others have also found influenza and RSV detections to be associated with syndromic surveillance data for respiratory illnesses, including ED visits among children,^20^ calls to a telehealth service,^21^ visits to general practitioners and hospitalizations.^15^ Of note, in our analysis, we found it important to allow for delays between the syndromic surveillance system and the laboratory data because unlagged laboratory detections (i.e., laboratory detections on the same day as ED visits) often had lower correlations with ED visits than detections a few days later. It makes sense that laboratory results would lag behind emergency department visits as factors such as sample collection, laboratory analysis and reporting will result in some delay. Future studies in this area should consider examining lagged test results as we have done here.

In contrast to non‐pandemic years, ILI‐related visit volumes during the Christmas–New Year holidays were lower during pandemic years, with the lowest holiday volumes in 2009–2010 following the second wave of the H1N1 pandemic. This lower volume cannot be explained by pandemic influenza response planning because influenza assessment centres were only open in Edmonton from October 30‐November 23, 2009^22^; therefore, volumes were not artificially decreased for this reason. Most laboratory‐confirmed influenza isolates during the 2009–2010 season in Canada were the pandemic H1N1 strain.^23^ Greater population immunity to this circulating viral strain due to prior infection in the first or second H1N1 pandemic wave (which occurred before the Christmas–New Year holiday period in 2009–2010) or vaccination during the pandemic may help to explain the reduction in holiday ILI‐related ED visit volumes this season.

Our analysis shows that the timing of the holiday surge in ILI‐related ED visits is highly predictable. As other Canadian research has recommended,^24^, ^25^ peaks in healthcare use such as this should be expected and prepared for. One potential option is to increase access to primary care during holidays,^24^ which may be successful, but challenging to implement. Another, less systematic, option may be to open dedicated influenza care clinics to improve access for patients with milder respiratory illnesses who do not require care at the ED, as was done in Edmonton in 2014. If this option is considered, our results suggest that it may be most effective if its timing coincides with the December 26 to January 2 time period.

This study has several limitations. First, it is an ecologic study, and we were unable to link ILI‐related ED visits with laboratory samples on an individual level. Such a study would provide a better understanding of infections experienced by those making ILI‐related ED visits. Second, in Part 1, we limited ILI‐related ED visits to those made by Edmonton residents and expanded the geographic scope of the laboratory data to submissions from a larger area to allow us to include more laboratory data. Third, by limiting ILI‐related ED visits to Edmonton residents, the true visit volume to the healthcare facilities included in our analysis is higher than we have reported: Edmonton residents made up approximately 63% of ILI‐related ED visits to the hospitals included in our analysis. Furthermore, we did not have access to physician billing data; therefore, we do not have full picture of healthcare use during the holidays and cannot see if, for example, during the holidays, the increase we observed in ILI‐related ED visits is coincident with a decrease in ILI‐related visits at physicians' offices; however, this is an area for future investigation. Fourth, during the H1N1 pandemic in 2009 and the 2013–2014 influenza season, public health actions, including the opening of influenza care clinics, were in place to reduce volumes at the ED; therefore, visit volumes are likely artificially decreased during these times as patients visiting these clinics were not recorded in these data (ARTSSN, personal communication, 24 November 2015). Finally, these data are affected by biases inherent in the healthcare seeking behaviour of the population and physician testing behaviours.

EDs experience an intense and highly predictable surge in ILI‐related visits during the Christmas–New Year holidays. Although the magnitude of this peak differs by season, it always represents the highest daily ILI‐related visit volume during non‐pandemic seasons and its timing occurs during the same 11 days. This predictability can enable emergency departments to prepare (e.g. by adjusting staffing) and for health authorities to potentially prevent or mitigate this increase in healthcare use (e.g. through dedicated influenza assessment centres). The ILI‐related ED visits examined were associated with detections for respiratory infections and most strongly associated with influenza A; however, the timing of the highest peak in respiratory virus detections is not always consistent with this peak in ILI‐related ED visits. Therefore, interventions beyond disease prevention strategies are likely needed to mitigate holiday pressure at the ED.
